# Preoperative Identification and Management of Skin Disorders Before Total Hip Arthroplasty: Two Case Reports and a Review of the Literature

**DOI:** 10.7759/cureus.115945

**Published:** 2026-09-08

**Authors:** Margarita-Michaela Ampadiotaki, Lamprini G Agapitou, Kalliopi Lampropoulou-Adamidou, Stavros Lykos, Aristidis Tsalkitzis, Charikleia Komari, Stamatios A Papadakis

**Affiliations:** 1 2nd Department of Orthopeadics, KAT General Hospital of Attica, Athens, GRC

**Keywords:** periprosthetic joint infection, staphylococcus epidermidis, staphylococcus lugdunensis, staphylococcus simulans, surgical site infection, total hip arthroplasty

## Abstract

Periprosthetic joint infection (PJI) is among the most devastating complications associated with total hip and knee arthroplasty (THA and TKA). Several risk factors for PJI have been identified; however, the bacterial skin flora at the surgery site has been underestimated. We present two cases of early identification of skin infection from *Staphylococcus lugdunensis, Staphylococcus simulans* and *Staphylococcus epidermidis* before THA, postponement of the surgery and targeted treatment of skin infection before rescheduling of THA. Four and three years after THA, patients remain without complications. The successful preoperative identification of skin inflammatory lesions around the hip led the surgeon to postpone the THA and treat them following consultation with an infectious disease specialist and a dermatologist. Careful preoperative skin preparation and observation by the surgeon are necessary. A review of the existing literature on these species as pathogens of PJI and the development of PJI in patients with skin disorders was conducted.

## Introduction

Periprosthetic joint infection (PJI) is among the most devastating complications associated with total hip and knee arthroplasty (THA and TKA), occasionally resulting in fatal outcomes. Its reported incidence in the literature ranges around 1-2% which is increasing over time [1]. PJIs provoke increased mortality and morbidity, rehabilitation delay, prolonged hospital stay and additional health care costs. The clinical management is based on criteria according to the Musculoskeletal Infection Society (a standardised set of clinical and laboratory findings used to objectively diagnose or rule out a PJI) [2]. Infections associated with implanted materials are particularly challenging to treat because biofilm formation (a protective matrix secreted by bacteria that adheres to prosthetic surfaces) renders them highly resistant to both antibiotics and the host's immune system [2].

Several risk factors for PJI have been thoroughly identified [3]. However, while existing guidelines mandate delaying elective arthroplasty for overt, active skin infections like cellulitis, they remain completely silent regarding non-inflammatory findings such as comedones on the incision line. In daily practice, these benign-looking keratin plugs are routinely underestimated as harmless cosmetic variants, presenting a significant clinical dilemma on whether to proceed or postpone surgery. This underrecognised blind spot overlooks the fact that comedones can act as deep, encapsulated bacterial reservoirs where standard surface antisepsis fails.

Therefore, this study presents two rare cases where the intraoperative identification of surgical-site comedones led to THA postponement. This report addresses a critical knowledge gap, demonstrating that comedones should not be ignored but managed via a multidisciplinary approach involving orthopaedic surgeons, dermatologists, and infectious disease specialists for localised clearance prior to implant placement. A review of the literature regarding these pathogens and PJI development in patients with skin disorders follows.

## Case presentation

Case 1

A 72-year-old Caucasian male presented at our outpatient clinic complaining of pain and stiffness in the left hip, which had deteriorated over the last year. Physical examination revealed limitation of both passive and active range of motion. Moreover, anteroposterior and lateral pelvis radiographs were performed, which showed severe osteoarthritis of the hip. As conservative treatment was ineffective, a THA was decided. His past medical history was recorded, including comedonal acne at a young age.

Showering with chlorhexidine gluconate soap the night before surgery was recommended. Preparation with hair removal from the site of the incision was performed by an orderly with an electric clipper with a disposable, single-patient-use head, and povidone-iodine and isopropyl alcohol were used for pre-operative skin preparation and sterilisation. Following skin preparation and sterilisation, multiple open and closed comedones became evident directly within the planned surgical incision site. Similar lesions, characterised by clustered dilated follicular openings with dark keratin plugs, were also present on the patient's face, neck, abdomen, and groin. The contents of one open and one closed comedone located at the incision site were aseptically collected for microbiological culture (Figure 1).

**Figure 1:**
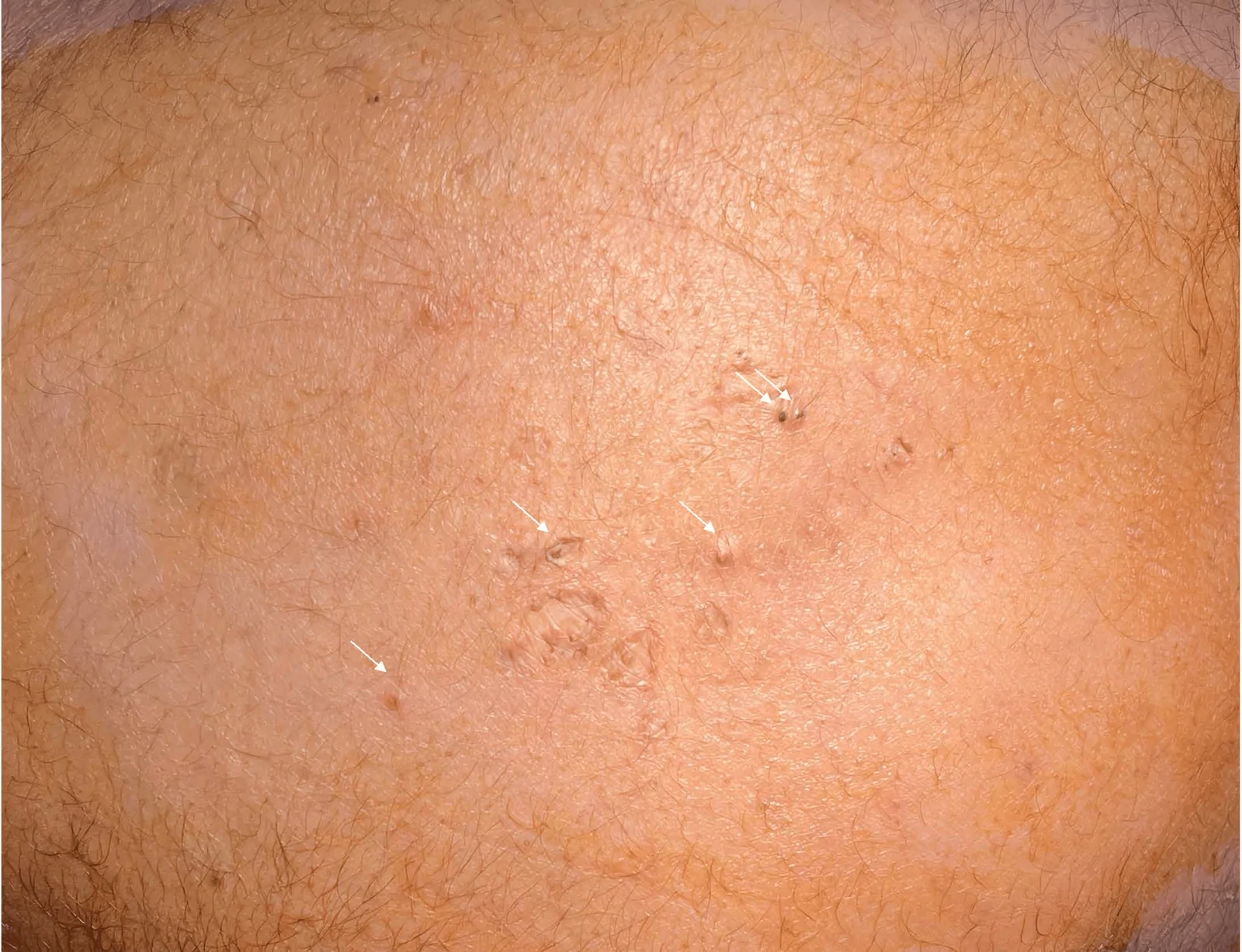
Comedones at the site of the incision inspected, aseptically collected, and cultured in Case 1. The arrows indicate open and closed comedones adjacent to the planned surgical incision site.

At that stage, immediately before incision, the decision to postpone the surgery was made, considering that the presence of an active skin infection could potentially increase the risk of PJI if the patient was undergoing an elective THA. Cultures showed two coagulase-negative staphylococci (CoNS, a broad group of bacteria that are normal components of the skin flora but can become opportunistic pathogens on medical implants): *Staphylococcus lugdunensis* and *Staphylococcus simulans* in both samples.

Based on the antibiogram and following consultation with an infectious disease specialist and a dermatologist, the patient was treated with 800 mg sulfamethoxazole and 160 mg trimethoprim twice a day orally for two weeks. Following the initial treatment, repeat specimens were obtained for microbiological culture. Although *Staphylococcus epidermidis* was isolated, it was considered part of the normal skin microbiota, and the surgery was subsequently rescheduled. Laboratory investigations, including the white blood cell count, C-reactive protein level, and erythrocyte sedimentation rate, were all within normal limits. Four years after THA, the patient remains without complications.

Case 2

A 55-year-old Caucasian male was scheduled for THA because of symptomatic coxarthrosis for more than six months. His medical history revealed hypertension, dyslipidemia and well-controlled Behcet disease with prednisolone 15 mg/d, colchicine and hydroxychloroquine. Cutaneous manifestations of his disease, such as papulopustular lesions characterised by folliculitis, comedones and acne-like papulopustules and healed ulceration lesions, were observed on the hip and the lower extremity.

The same skin preparation as in Case 1 was performed the night before surgery and in the theatre before the skin incision. Comedones directly at the site of the incision became evident, collected aseptically and cultured (Figure 2).

**Figure 2:**
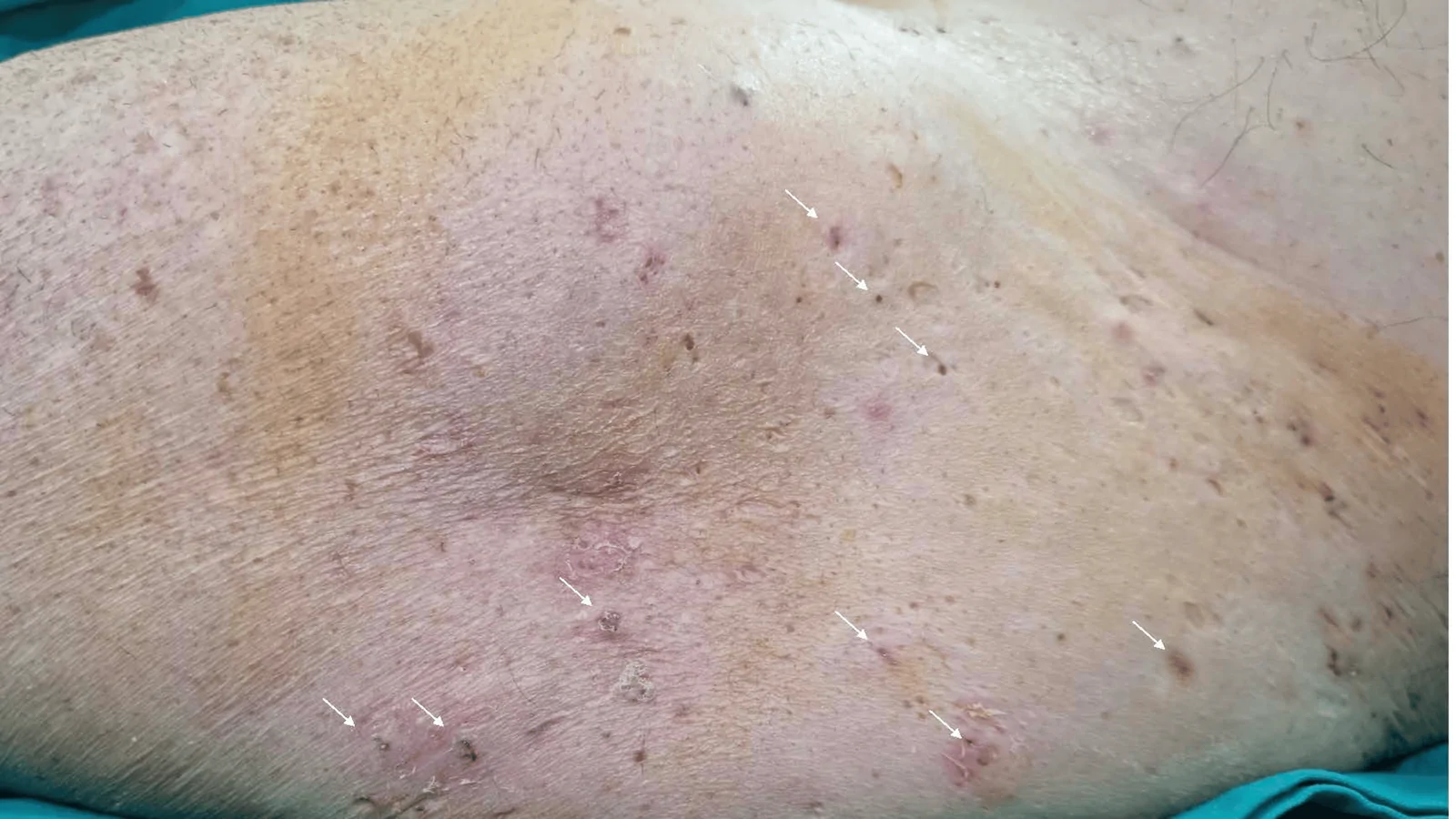
Comedones at the site of the incision inspected, collected aseptically and cultured in Case 2. The arrows indicate open and closed comedones adjacent to the planned surgical incision site.

As in Case 1, immediately before incision, the surgery was postponed due to concerns that an active skin infection could increase the risk of PJI following elective THA. *Staphylococcus epidermidis*, which was isolated, is considered a member of the skin microbiome. For this reason, it was decided that the patient did not require oral antibiotic treatment. The surgery was rescheduled after local treatment of the comedones with a CO_2_ laser. Three years after the THA, the patient remains without complications.

## Discussion

Periprosthetic joint infection prevention requires a combination of pre-, intra- and post-operative measures such as preoperative tobacco cessation and treatment of any infection in the patient, preoperative skin antisepsis, surgical hand antisepsis, the use of sterile gowns, appropriate surgical attire, antibiotic-impregnated cement, and antibiotic prophylaxis. According to the existing literature, common isolated species of pathogens are: *Staphylococcus*, *Streptococcus*, *Enterococcus*, *Pseudomonas* and *Escherichia coli* [4].

The proliferation of keratinocytes produces the formation of comedones. Several factors have been described in comedogenesis, including abnormalities in lipid composition, androgens, cytokine production and bacteria. Cultures of comedones have shown that about 30% of them are bacterial-free. Although comedones undergo cyclic growth, which lasts between two to six weeks, they may refill during this period [5]. Samples from acne lesions such as comedones revealed the development of many different species. These species include *Staphylococcus*, *Streptococcus*, *Pseudomonas*, *Klebsiella*, *Enterobacter *and *Proteus *species, in addition to the well-known *Propionibacterium acnes*, which are all pathogens of PJI as well [6].

Published dermatological data indicate that the vast majority of comedones contain high bacterial loads; however, orthopaedic literature has overlooked them. In routine arthroplasty practice, comedones may be perceived as inert cosmetic features rather than microbiological reservoirs. Because they lack classic inflammatory signs like surrounding erythema or warmth, a surgeon could normally proceed with the scheduled THA. However, our findings confirm that these plugged follicles seal bacteria deeply, effectively shielding them from standard preoperative chlorhexidine skin preparation. It becomes evident that non-inflammatory comedones represent a major, unaddressed risk factor.

Some studies revealed that even if the incision is made on apparently healthy skin of patients with dermatitis, especially atopic, even at a distant location, the risk of implant infection remains high [7]. On the other hand, authors reporting a successful THA in a patient suffering from epidermolysis bullosa encourage surgeons to perform surgeries on patients with dermatological diseases after considering adequate infection control [8]. Furthermore, TKA in patients suffering from psoriasis had no significant difference in infection rates according to Beyer et al. [9]. However, a recent study showed a three-fold increase in risk of PJI in patients with psoriasis [10].

*Staphylococcus lugdunensis* (SL), which was isolated in Case 1, was initially described in 1988 as a cause of serious infections, such as endocarditis and peritonitis [11]. It is a part of the normal skin flora and is usually found in the perineum and inguinal area. In 2000, Sampathkumar et al. described periprosthetic joint infections from SL for the first time [12]. A large case series that studies staphylococci as pathogens in PJIs suggests that SL share many properties, such as the clinical picture and the high pathogenicity, with *Staphylococcus aureus* in contrast with *Staphylococcus epidermidis*. However, SL differs from *Staphylococcus aureus* in antimicrobial susceptibility [13]. According to the literature, an appropriate treatment is the combination of fluoroquinolone and rifampicin [14], while treatment with a beta-lactam is more successful than vancomycin [15]. A recent multicentric retrospective cohort that includes 111 patients with proven SL PJI pointed out the high rate of treatment failure (around 20%) and concluded that this is a difficult-to-treat infection [16]. Table 1 presents studies that found PJIs due to SL; discrepancies in the outcomes directly reflect the expected heterogeneity of the current literature [13,17,18].

**Table 1:** Published studies reporting periprosthetic joint infections due to Staphylococcus lugdunensis. M, male; N/A, not applicable; DAIR, debridement, antibiotics and implant retention. * Of reported cases.

Author (year)	Cases	Gender	Age, years (range)	Outcome	Surgical treatment	Treatment	Joint replacement
Adl Amini et al. (2023) [18]	5 (10.9%*)	N/A	N/A	One recurrent infection (20%)	100% two-stage revision	N/A	Hip
Lourtet-Hascoët et al. (2016) [13]	28	46% M	68 (58-78)	89% without relapse for at least a year	39% two-stage revision, 21% one-stage rev, 39% DAIR	Fluoroquinolone/rifampicin or linezolid or oxacillin for a median of 7 weeks	10 Hip (36%), 16 knee (57%), 1 shoulder (4%), 1 foot (4%)
Masood et al. (2020) [17]	28	Ν/Α	Ν/Α	Relapse: 15% after two-stage revision, 44% debridement, 100% no surgical intervention or one-stage revision	46% two-stage revision, 4% one-stage rev, 32% DAIR, 21% no surgery	Οxacillin	Hip (25%), knee (68%), other (7%)
Shah et al. (2010) [15]	28 (22 patients)	50% M	74 (35-88)	85% without relapse after 24 months (86% two-stage revision and 79% after DAIR)	50% two-stage revision, 4% one-stage rev, 36% DAIR, 4% resection arthroplasty, 7% no surgery	Cefazolin (n=11) or ceftriaxone (n=3) or vancomycin (n=13) IV for a median of 38-39 days, followed by PO antibiotics	Hip (11%), knee (89%)
Sampathkumar et al. (2000) [12]	1	M	72	No relapse 10 months after reimplantation	Two-stage revision	Ceftriaxone IV for 6 weeks	Knee
Sampathkumar et al. (2000) [12]	1	M	74	No relapse 8 months after DAIR	DAIR following reimplantation before 6 weeks	Cefazolin was administered for 6 weeks, followed by long-term PO suppression with cephalexin	Knee

*Staphylococcus simulans* attracts less attention in comparison to staphylococcal species that are isolated from clinical specimens more frequently, such as *Staphylococcus epidermidis* and *Staphylococcus lugdunensis* [10]. *Staphylococcus simulans* is a CoNS and constitutes a common pathogen among animals. Human infections from *Staphylococcus simulans* are extremely rare, including endocarditis, osteomyelitis, pneumonia, and urinary tract infections. Generally, a few cases of orthopaedic infections, including PJIs, due to *Staphylococcus simulans* have been described [19]. An in vitro study examined the pathogenicity of CoNS, and none of the examined *Staphylococcus simulans* produced biofilms. Nevertheless, some staphylococci of this species had the potential to generate biofilm in other studies [10].

In the second case mentioned above, cultures revealed *Staphylococcus epidermidis*. The evaluation of clinical samples for *Staphylococcus epidermidis* can be challenging because this species is commonly found in normal skin flora. Additionally, its subspecies often exhibit significant variability, making it difficult to distinguish between contamination and actual infection. Recent evidence indicates that the pre-admission microbiota of certain patients may harbour *Staphylococcus* lineages associated with an increased risk of periprosthetic joint infection (PJI). Thus, a preoperative shower with chlorhexidine gluconate soap is recommended, and broader antimicrobial prophylaxis has been suggested in order to reduce the incidence of PJI due to these species [20]. As observed in our second case, the presence of comedones may be associated with an increased risk of *Staphylococcus* infection. Hence, careful skin observation by the surgeon immediately before surgery is necessary.

The microbiological findings directly influenced our perioperative management. Isolating the specific pathogens enabled the administration of targeted, susceptibility-guided antimicrobial therapy rather than empirical treatment. Ultimately, these cases modified our subsequent clinical practice, confirming that encountering comedones directly on the planned incision line necessitates temporary deferral of the elective arthroplasty, microbiological screening, and targeted local treatment before rescheduling the surgery as a staged procedure.

Although numerous studies have evaluated PJI pathogens and established patient-related risk factors, there is no evidence regarding the management of localised skin lesions located directly within the planned surgical incision immediately before elective arthroplasty. Consequently, rescheduling of the surgery was safely decided following the completion of the appropriate antimicrobial therapy. Finally, recommendations regarding microbiological assessment and surgical decision-making in these situations remain largely based on expert opinion rather than clinical evidence. Our cases therefore contribute by illustrating a practical management strategy in this uncommon clinical scenario rather than establishing a causal relationship between these skin lesions and subsequent PJI.

## Conclusions

Current orthopaedic guidelines lack clear protocols regarding non-inflammatory skin lesions, such as comedones, at the surgical site. Arthroplasty surgeons must recognise that these benign-looking keratin plugs frequently harbour hidden bacterial loads that escape standard surface skin antisepsis. Encountering comedones directly along the planned incision line should not be dismissed as a minor cosmetic variant. Based on our experience, we advocate that such findings require a high index of suspicion, temporary postponement of elective total joint arthroplasty, and a multidisciplinary approach involving dermatologists and infectious disease specialists to ensure targeted systemic and local treatment and safely eliminate an unrecognised reservoir of infection before implant placement.
